# The fusion rate of demineralized bone matrix compared with autogenous iliac bone graft for long multi-segment posterolateral spinal fusion

**DOI:** 10.1186/s12891-015-0861-2

**Published:** 2016-01-05

**Authors:** Tsai-Sheng Fu, I-Chun Wang, Meng-Ling Lu, Ming-Kai Hsieh, Lih-Huei Chen, Wen-Jer Chen

**Affiliations:** Department of Orthopaedic Surgery, Chang Gung Memorial Hospital in Keelung, School of Medicine, Chang Gung University, Taoyuan, Taiwan; Department of Orthopaedic Surgery, Chang Gung Memorial Hospital in Linkou, School of Medicine, Chang Gung University, Taoyuan, Taiwan

**Keywords:** Multi-segment posterolateral fusion, Demineralized bone matrix, Bone graft substitute, Autogenous iliac bone graft, Osteoconduction, Osteoinduction, Osteogenesis

## Abstract

**Background:**

Although autogenous iliac bone graft (AIBG) remains the gold standard for spine fusion, harvesting morbidity has prompted the search for alternatives especially for multi-segment fusion. This study aimed to evaluate the efficacy of using demineralized bone matrix (DBM) as a substitute of AIBG for long instrumented posterolateral fusion (≧ three-level fusion).

**Methods:**

A total of 47 consecutive patients underwent laminectomy decompression, and multi-level instrumented posterolateral fusions were reviewed. Group 1 comprised 26 patients having DBM with autologous laminectomy bone (ALB). Group 2 consisted of 21 patients having AIBG with ALB. The fusion success evaluation was based on findings using the 12-month anteroposterior and dynamic plain radiographs.

**Results:**

Gender, age, and the number of fusion levels were similar for both groups. 21 of 26 (80.8 %) patients in group 1 and 18 of 21 (85.7 %) patients in group 2 were observed to achieve solid bony fusion. There was no statistical difference in the fusion success (*p* = 0.72). Blood loss was significantly more in group 2 (*p* = 0.02). The duration of the hospital stays and operative times being longer for group 2, but the difference was not significant.

**Conclusions:**

DBM combined with ALB and osteoconductive materials is as effective as an autologous iliac bone graft with respect to long multi-segment posterolateral fusion success. DBM can be used as an effective bone graft substitute and may decrease morbidities associated with iliac bone graft harvest.

## Background

Successful surgical management of spine instability often requires bone grafting for fusion. An ideal bone graft has three components for bone formation: osteoconduction, osteoinduction, and osteogenesis. Autogenous iliac bone graft (AIBG) remains the gold standard for successful spine fusion. However, in addition to the problem of limited quantity, harvesting morbidity has prompted the search for suitable alternatives [1, 2]. Currently, there are many different types of bone graft substitutes available and under development. Recombinant growth factors, bone morphogenetic proteins (BMPs), demineralized bone matrix (DBM), ceramics and collagen-based matrices function as osteoinductors or osteoconductors are gaining popularity and are being increasingly used in the lumbar spine. Through chemical and demineralized processing, DBM preserves the natural capacity of the native bone proteins and growth factors [3].

For one- or two-level short instrumented posterolateral fusion, our previous studies showed that autologous laminectomy bone (ALB) with synthetic osteoconductive material achieved high fusion success [4]. However, Steffen et al. reported that posterolateral fusions demand approximately 15 mL of compacted bone per fused level per side [5]. A greater quantity of bone graft is needed for multi-segment long posterolateral fusion success. This quantity cannot typically be obtained from locally harvested ALB. Theoretically, in combination with ALB, DBM, and synthetic osteoconductive materials, these composites can provide all three components of bone formation. Thus, an osteoconductive bone graft substitute and DBM can be used to expand an existing quantity of available local laminectomy bone chips for the purpose of multi-segment long posterolateral fusion.

Several studies demonstrated its clinical effectiveness of DBM in bone formation for long bone defect application, devastating digits bony injury, and craniofacial bone injury [6–9]. To our knowledge, there are no published data about DBM used for multi-segment spine fusion. The purpose of this study was to evaluate the clinical and radiographic performance of using DBM and osteoconductive bone graft substitute as an extender to ALB for instrumented multi-segment long posterolateral spine fusions.

## Methods

After obtaining approval from the Institutional Review Board (IRB) of Chang Gung Medical Foundation (reference number: 103-5000B) without the need for informed consent due to retrospective study, 47 consecutive adult patients (11 males and 36 females), who underwent decompression and multi-level pedicle screw instrumented posterolateral fusion (≧ three-level fusion) between January 2009 and December 2013 were retrospective reviewed. The mean patient age was 66.3 ± 8.3 years old (range: 52–87 years old). Medical charts were reviewed, including diagnosis, surgical procedures, and postoperative complications. All the cases were diagnosed and treated based on clinical symptoms, plain radiographs, and magnetic resonance imaging studies. After the failure of initial conservative treatments, surgery was performed as a treatment option. The indications for surgery included lumbar spinal stenosis with spondylolisthesis and scoliosis (*n* = 41) and failed earlier back surgery (*n* = 6). All the patients underwent laminectomy for nerve decompression with pedicle screw instrumentation and posterolateral fusion including three-level instrumentation and fusion in 24 patients, four-level instrumentation and fusion in 20 patients, five-level instrumentation and fusion in 1 patient, six-level instrumentation and fusion in 1 patient, and seven-level instrumentation and fusion in 1 patient. The patients were divided into two groups. Group 1 comprised 26 consecutively operated patients (6 males and 20 females with a mean age of 67.2 ± 9.4 years) in whom DBM was used. Group 2 (control group) consisted of 21 consecutively operated patients (5 males and 16 females with a mean age of 65.1 ± 6.7 years) operated on prior to the introduction of DBM in our department. Autologous iliac bone graft was used for group 2 patients. Twelve patients in group 1 and eight patients in group 2 underwent posterior lumbar interbody fusion (PLIF) or transforaminal lumbar interbody fusion (TLIF) for sagittal and coronal alignment correction. Gender, age, TLIF/PLIF procedures, and the number of fusion levels were similar for both groups (Table 1).

**Table 1:** Comparisons of demographic data between groups

	Group		Significance test
	DBM	AIBG	
	(*n* = 26)	(*n* = 21)	(*p*-value)
Age (years)	67.19 ± 9.42	65.10 ± 6.66	0.39
Gender (n, male/female)	6:20	5:16	1.00
TLIF/PLIF (n)	12	8	0.58
Operative levels (n)	13 three-level	11 three-level	0.58
	11 four-level	9 four-level	
	1 five-level	1 six-level	
	1 seven-level		

The continue data were compared with independent t test while the categorical data were compared with Chi-square test or Fisher’s exact test

*DBM* demineralized bone matrix, *AIBG* autogenous iliac bone graft, *TLIF* transforaminal lumbar interbody fusion, *PLIF* posterior lumbar interbody fusion

Posterior approaches were carried out utilizing the conventional standard open technique in every case. After meticulous stripping of all the covering soft tissues of the decompression site, a laminectomy was performed to remove the spinal process, lamina, and partial facet for nerve decompression. The ALB chips were extracted in small pieces and collected. The attached ligamentum flavum was also removed as well as possible. In order to have an adequate supply of ALB chips, the levels of laminectomy included all the fusion segments but preserved the half-spinal process and lamina of the top cranial and caudal vertebra. This ensured the preservation of the integrity of the posterior complex (spinal process/supraspinous and interspinous ligament/spinal process) between the fused segments and the neighboring motion segments to prevent future adjacent instability [10]. Autogenous iliac bone graft was harvested from the posterior iliac crest by opening an approximately 3 × 3 cm window at the outer cortex. About 10 mL autologous cancellous bone chips were harvested for grafting. The DBM bone grafts used for group 1 were 5 mL commercially available DBM putty (Allomatrix, Wright Medical Technology, Arlington, TN). The quantity of AIBG used for group 2 was around 10 mL. The DBM (group 1) and AIBG (group 2) were both mixed with hydroxyapatite-β-tricalcium phosphate granules (Foramic; Maxigen Biotech Inc., Taiwan) and ALB chips. The quantity of hydroxyapatite-β-tricalcium phosphate granules was according to the volume of ALB chips. It should not be more than the volume of ALB chips. After placing the transpedicular screws at the target levels and decortication of the transverse processes, the indicated fusion segments were joined by contoured rods. The surgical area was irrigated with normal saline before placement of the bone graft materials. The bone graft mixture was then placed over the decorticated bone on both sides. A negative-pressure drainage tube (Hemovac; Zimmer, Dover, OH) was used in all cases for an average of 3 days.

**Fig. 1:**
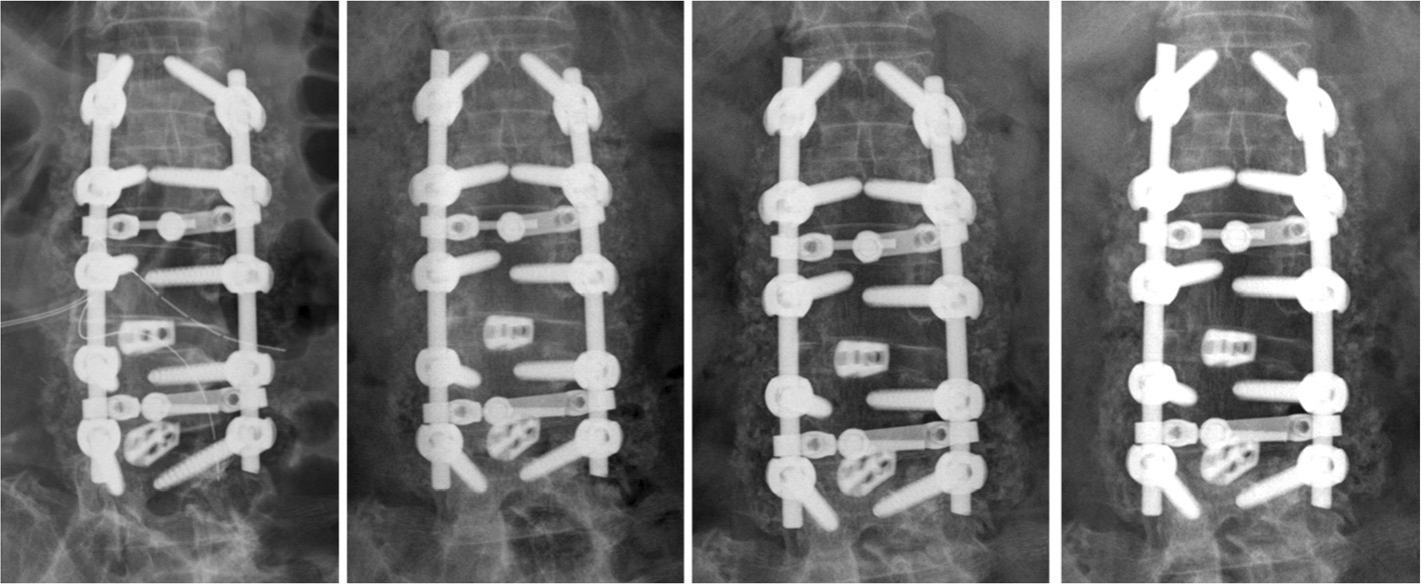
Radiographs from left to right showed the immediately, 3, 6, and 12 months postoperatively. The immediately postoperative image showed multiple opacity spots at the intertransverse space indicated a mixture of hydroxyapatite-β-tricalcium phosphate granules with laminectomy bone chips and DBM. The 12-month image showed a successful fusion mass with continuously qualitative bony bridging at the intertransverse fusion areas

**Fig. 2:**
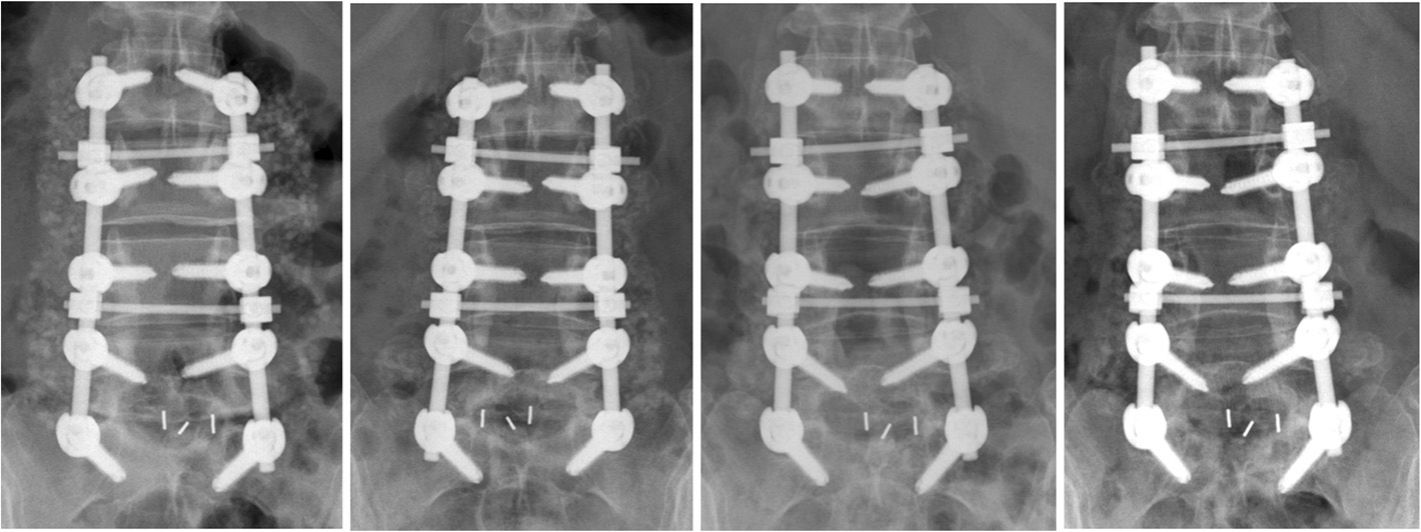
Radiographs from left to right showed the immediately, 3, 6, and 12 months postoperatively. The immediately postoperative image showed bone graft materials (tricalcium phosphate granules, laminectomy bone chips and DBM) at the intertransverse space. The subsequent images showed absorption of the bone graft materials and unsuccessful fusion mass formation

Follow-up was done on patients radiologically at 3, 6, 12 and 18 months after surgery. The fusion success evaluation was based on findings on the 12-month plain radiographs. Radiographic assessment of the fusion was based mainly on anteroposterior and flexion-extension dynamic plain radiographs because not every patient would consent to computerized tomography (CT) scans. Fusion criteria described by Christensen et al. were applied to evaluate all plain radiographs [11]. The fusion mass should be visible laterally to the instrumentation and at the intertransverse fusion areas. A successful fusion was determined as continuously qualitative intertransverse bony bridging at the target level on the follow-up radiographs (Fig. 1). Suboptimal quality or a fusion mass hidden by the instruments is considered as unsuccessful fusion (Fig. 2).

Statistical analyses were performed using the Statistical Package for the Social Sciences for Windows (SPSS, version 12.0; IBM, Armonk, NY, USA). For continuous variables, the independent t-test or the Mann–Whitney test were used to determine any significant difference between the groups. Fisher’s exact tests or Chi-square test were applied for categorical variables because the sample size is not enough. Statistical significance was set at a *p* value of less than 0.05.

## Results

**Fig. 3:**
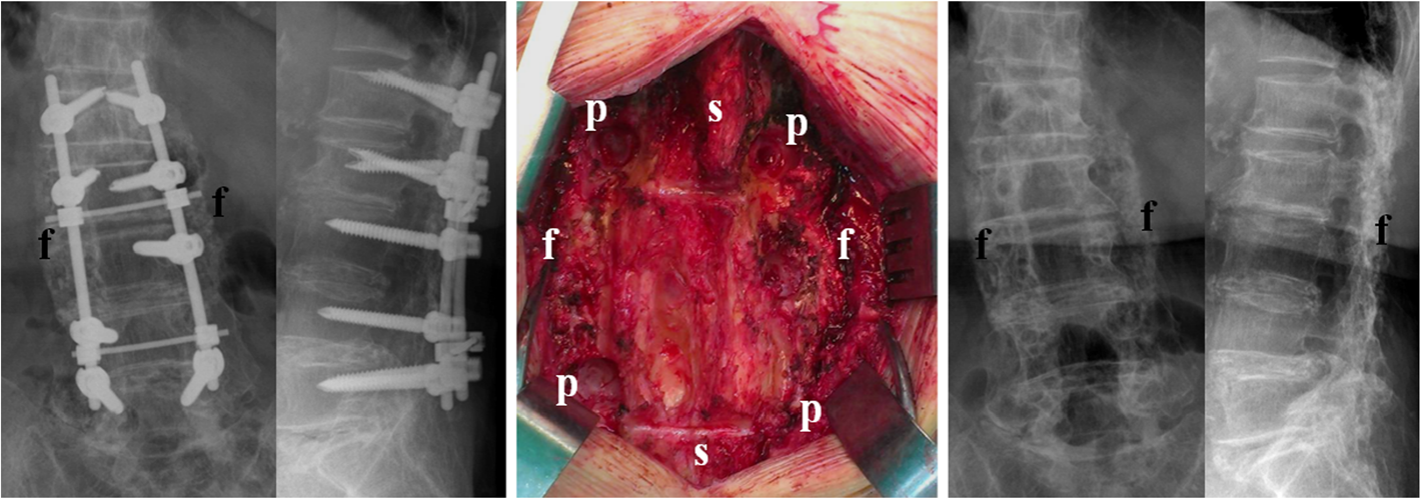
A 80-year-old female underwent instrumentation and posterolateral fusion with DBM from L1 to L5 and received second operation for removal of implants. (*Left*) The postoperative 12-month image showed successful fusion mass formation with pedicle screws loosening. (*Middle*) The intraoperative image showed solid and continuous fusion mass formation. (*Right*) After removal of the implants, the plain radiographs showed continuous fusion mass formation from L1 to L5. f, fusion mass; p, pedicle screw hole; s, spinal process

Table 2 lists the results. On the plain radiographs 12 months after surgery, 21 of the 26 patients (80.8 %) in group 1 were observed to achieve solid bony fusion; 18 of the 21 (85.7 %) patients in group 2 were observed to achieve solid bony fusion. The difference was not statistically significant (*p* = 0.72). The five cases showing unsuccessful fusion in group 1 were 1 three-level, 3 four-level, and 1 seven-level fusions. The three cases showing unsuccessful fusion in group 2 were 1 three-level and 2 four-level fusions. There were 11 cases (42.3 %) in group 1 and 6 cases (28.6 %) in group 2 that showed screw loosening on the last follow-up plain radiographs. The difference was not statistically significant (*p* = 0.33). The screw loosening did not result in worse posterolateral fusion success (Fig. 3).

**Table 2:** Comparisons of the results between groups

	Group		Significance test
	DBM	AIBG	
	(*n* = 26)	(*n* = 21)	(*p*-value)
Fusion/Non-fusion (n)	21/5	18/3	0.72
Operating time (min)	284.89 ± 62.40	304.62 ± 61.64	0.28
Blood loss (ml, median (IQR))	700 (450,950)	1200 (725,1675)	0.02^a^
Hospital stay (days)	10.46 ± 7.77	11.33 ± 6.58	0.68
Implant loosening (n)	11	6	0.33

The continue data were compared with independent t test and the Mann-Whitney test while the categorical data were compared with Fisher’s exact test

*DBM* demineralized bone matrix, *AIBG* autogenous iliac bone graft, *IOR* interquartile range

^a^Statistically significant. analysed by the Mann-Whitney test

The operating time for group 1 was 284.88 ± 62.40 min and for group 2 was 304.62 ± 61.64 min. There was no significant difference (*p* = 0.28). A significant difference (*p* = 0.02) was found between blood loss for group 1 (700 (450–950) mL) and group 2 (1200 (725–1675) mL). The hospital stay duration was longer for group 2 (11.33 ± 6.58 days) than group 1 (10.46 ± 7.77 days), although the difference was not significant (*p* = 0.68).

In group 1, two cases had deep wound infection. The infections were successfully treated by debridement for seven-level fusion case and by removal of spine implants for four-level fusion case. They both had long hospital stay due to received 6-week parenteral antibiotic treatment. In group 2, one case had a persistent fever after surgery. Wound infection was suspected and successfully treated with a 2-week empiric parenteral antibiotic without long-term consequences.

## Discussion

This study focuses on the safety and efficacy of an available DBM bone graft material used as a graft extender in multi-segment posterolateral instrumented lumbar fusion. Overall, the successful fusion rate was documented with an average of 80.8 %, which is comparable to the results with an autogenous iliac bone graft. The results demonstrate the benefits of composite DBM and osteoconductive material with a lamina local bone graft for multi-segment lumbar spine fusion. DBM could be successfully used as a bone graft extender for multi-segment posterolateral fusion success. Through the combination of DBM, autogenous laminectomy bone chips, and synthetic osteoconductive materials, the custom bone graft composites can provide all three components: osteogenesis, osteoinduction and osteoconduction, for bone formation. It can be used as an effect bone graft substitute for multi-segment posterolateral lumbar fusion and may decrease morbidities associated with autogenous iliac bone graft harvest.

AIBG remains the gold standard for successful spine fusion. However, it should be recognized as having associated complications such as donor site morbidity, postoperative pain, added blood loss, and increased surgical time [1, 2]. The current study demonstrated the same findings that the operative time, blood loss and hospital stay duration being higher in the autologous iliac bone graft group.

Autologous laminectomy bone is harvested from the lamina, spinous process, and facet during the decompression procedures. For single-level posterolateral fusion, ALB has similar successful fusion rates as AIBG [4, 12–16]. In contrast, ALB has worse results for multi-level fusions compared with AIBG [16]. This reflects the shortages of ALB, including its relatively limited quantity for multi-level fusions, and urges us to determine whether the DBM and synthetic osteoconductive material could be used as a bone expander in conjunction with ALB especially for multi-level posterolateral fusions.

DBM is a form of allograft created from cadaveric bone without the mineral content and the risk of disease transmission. The remaining type I collagen and non-collagenous proteins can serve as an osteoconductive scaffold [3]. It also has been demonstrated to be osteoinductive, or capable of inducing bone formation in heterotopic sites [17]. Compared to recombinant growth factors, it is relatively less expensive and unlimited in quantity. DBM has some shortcomings, including its highly variable osteoinductive properties and possible nephrotoxicity in animal studies [3]. The products of different manufacturing companies, even of the same manufacturer, may contain variability of growth factors for osteoinductive properties. Bae et al. found significant lot-to-lot variability in bone morphogenetic protein concentrations, which resulted in variable rates of fusion in vivo [18]. In the current study, the authors did not use DBM alone for posterolateral fusion. All lots of commercially available DBM putty are tested for inductivity prior to release by manufacturing company. The testing methods have been correlated with new bone formation in athymic rat models, which is widely considered the gold standard for assessing the osteoiductivity of a material [19].

In addition, the hydroxyapatite-β-tricalcium phosphate granules used in the current study also provided the property of the osteoconductive scaffold that facilitated the fusion mass formation. In our previous study and as other literatures showed, the calcium sulfate or hydroxyapatite-β-tricalcium phosphate granules combined with ALB provided equivalent bone formation and fusion success to AIBG for one- or two-level short instrumented posterolateral fusion [4, 20]. These osteoconductive materials resorb completely as newly formed bone remodels and allow blood vessels and osteogenic cells in-growth [21]. ALB is an autogenous bone graft harvested from the posterior elements in the spine structure including lamina, facet joint and spinal processes. Theoretically, it may provide osteogenic cells and osteoinductive and osteoconductive functions although the ALB bone chips are mainly cortical in nature. The DBM provides osteoinductive and osteoconductive functions. The hydroxyapatite-β-tricalcium phosphate granules provide osteoconductive function. In combination with ALB, DBM and synthetic osteoconductive materials, these composites may provide an adequate amount of bone graft with all three components of bone formation for long multi-level posterolateral spine fusion.

This study had some limitations. First, using plain radiographs alone to determine the status of posterolateral spine fusion is still questionable. The use of CT scans with sagittal, coronal and 3-dimensional reconstruction is suggested to improve the accuracy of fusion evaluation. However, using CT scans increases the amounts of radiation to which the patient is subjected and not every patient would consent to CT scans examination. Second, the current study is retrospective and not randomized. The sample size is relatively small and not large enough to detect the difference. However, the present study has the advantage of consistency between the two groups. The included patients were in a consecutive series before and after the availability of DBM in our hospital. The two groups examined here appear comparable in terms of the fusion success since the demographic data between groups were similar. All the operative procedures and follow-ups were performed at a single institution and by the same surgeon, this study eliminated possible differences in surgical technique and had excellent compliance with follow-up. Since all the patients had been followed up on for at least 18 months, we could use the 12-month plain radiographs for fusion status evaluation and comparison.

## Conclusions

The results of current study demonstrated that DBM combined with autologous local lamina bone chips and synthetic osteoconductive materials is as effective as autogenous iliac bone graft for the purposes of long multi-segment posterolateral fusion success. The custom bone graft composites can provide all three bone-formation components: osteogenesis, osteoinduction and osteoconduction. DBM can be used as an effective bone graft substitute for posterolateral lumbar fusion and may decrease morbidities associated with iliac autograft harvest.

## Abbreviations

AIBG: Autogenous iliac bone graft
DBM: Demineralized bone matrix
ALB: Autologous laminectomy bone
BMPs: Bone morphogenetic proteins
IRB: Institutional Review Board
CT: Computerized tomography
PLIF: Posterior lumbar interbody fusion
TLIF: Transforaminal lumbar interbody fusion
IOR: Interquartile range
